# Leishmaniasis vaccine research: current status and new directions

**DOI:** 10.1017/S0031182025101522

**Published:** 2026-03

**Authors:** Derya Topuz Ata, Anıl Ata, Chris (Katharine C.) Carter

**Affiliations:** 1 Department of Pharmaceutical Microbiology, Ankara University Faculty of Pharmacy, Ankara, Turkey; 2 Department of Biochemistry, Ankara University Faculty of Pharmacy, Ankara, Turkey; 3 Strathclyde Institute of Pharmacy and Biomedical Sciences, University of Strathclyde, Glasgow, UK

**Keywords:** clinical trials, CRISPR-cas9 technology, *Leishmania*, vaccines

## Abstract

Leishmaniasis is a neglected parasitic disease responsible for significant morbidity and mortality. Currently, there is no vaccine approved for clinical use. Therefore, controlling infections in infected individuals depends on interventions to prevent infected female sand flies from biting humans, treatment of clinical infections or alternative treatment methods. This review focuses on the types of vaccine developed to control leishmaniasis and which vaccines have made it through to clinical trials. It also discusses the role CRISPR technology may play in improving vaccine candidates design.

## Graphical abstract

**Figure:**
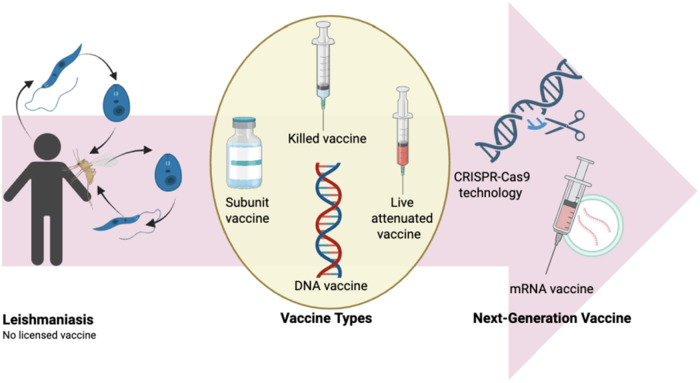

## Introduction

Leishmaniasis is a vector borne infection transmitted via the bite of a female sand fly infected with the protozoan parasite *Leishmania*. It is estimated that there are 700,000 to 1 million new cases each year and that 350 million people are at risk (Mehrotra et al., 2025). The symptoms presented by patients in the clinic depend on the infection species, vector prevalence, and host and parasite factors (Corman et al., 2023). There are over 20 different *Leishmania* spp. that cause infections in humans and infections can be characterized as three main types, i.e. cutaneous leishmaniasis (CL), visceral leishmaniasis (VL), and mucocutaneous leishmaniasis (MCL; Volpedo et al., 2021). CL is associated with the development of skin lesions, which can be singular or multiple and is caused primarily by infection with *L. major, L. tropica* or *L. braziliensis.* VL, caused by infection with *L. donovani* or *L. infantum*, is associated with parasites residing in the liver and spleen, resulting in hepatosplenomegaly, and can be fatal if untreated (Mahor et al., 2023). MCL, caused by infection with *L. braziliensis*, is a form of CL where the parasites disseminate to the mucous membranes, causing considerable damage to the face, which can result in significant disfigurement (Fischer et al., 2024). Factors such as concentrated populations, malnutrition and poor sanitation encourage disease transmission therefore, leishmaniasis is often viewed as a disease associated with poverty (Cosma et al., 2024). Control of leishmaniasis currently depends on preventing infected sandflies from feeding on humans e.g. by using insecticide impregnated bed nets (Montenegro-Quiñonez et al., 2022), killing the vector using insecticides (Kumari et al., 2025), or treatment of clinical cases using drug treatment or alternative methods such as cryotherapy, heat treatment (Shmueli and Ben-shimol, 2024). There is a limited number of drugs available to treat leishmaniasis and the current drugs have a number of drawbacks, including high toxicity, long treatment regimens, administration via parenteral routes in a hospital setting, ability to induce drug resistance and high costs (Singh et al., 2024). Ideally, a vaccine is required which is safe to use, induces a high level of protection against infection with one or more *Leishmania* spp., is affordable for people in *Leishmania* endemic countries, and can be given by a non-invasive route (Kaye et al., 2023b). The type of immune response associated with protection is species-specific but ultimately it must induce killing of the intracellular amastigote within the infected macrophage or killing the infected macrophages (Aruleba et al., 2020).

## The life cycle of *Leishmania*

**Figure 1.**
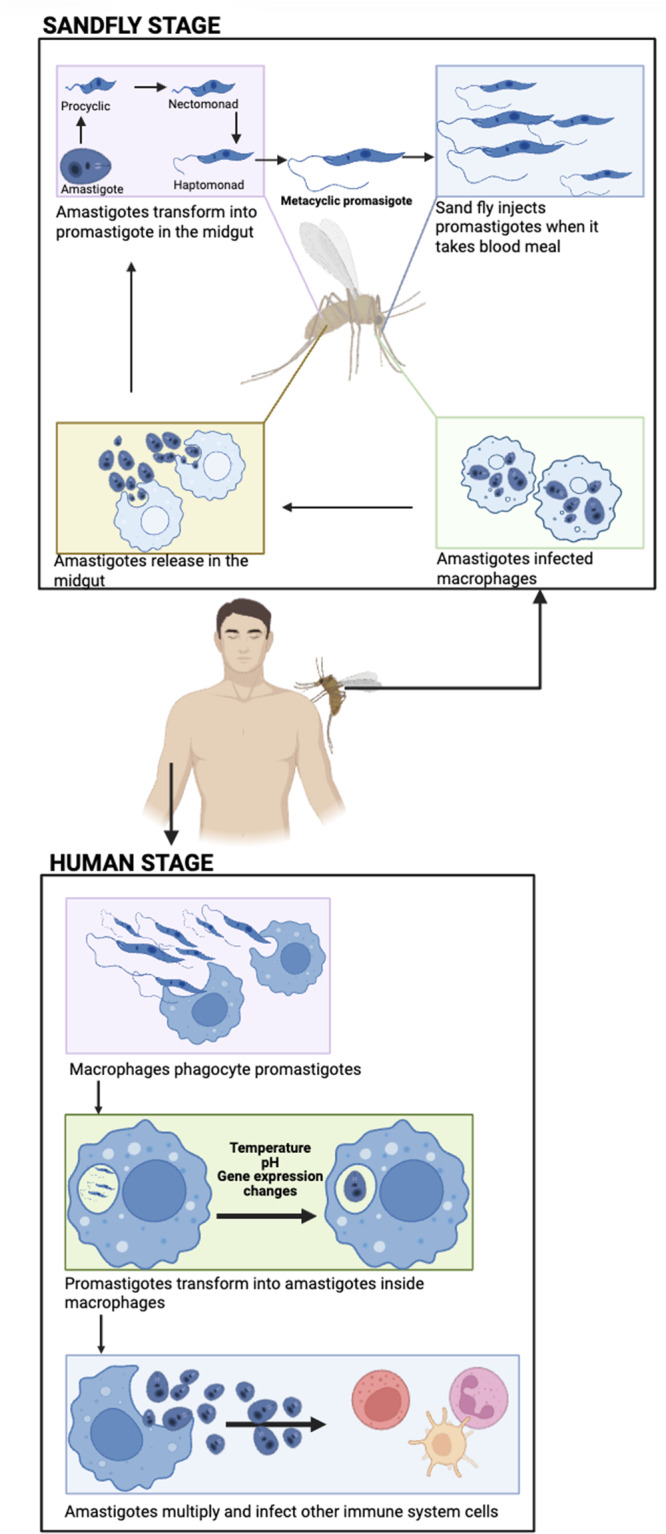
Life cycle of Leishmania. A sandfly deposits infective, metacyclic promastigote into the skin of a human during a bloodmeal. The promastigote parasites are taken up by phagocytic cells and transform into amastigotes form. The amastigotes stage multiply within the infected cells, and when the cell bursts amastigotes are released into the bloodstream. These parasites can infect new phagocytic cells or they can be taken up by a sandfly when it takes a bloodmeal from the infected individual. The amastigotes transform into procyclic promastigotes, which undergo a series of developmental changes until they mature into metacyclic promastigotes in the sandfly. The metacyclic promastigotes form a plug in the anterior midgut, ensuring that the sandfly regurgitates the parasites during feeding (created using BioRender.Com software). Figure 1 long description.

*Leishmania* has two main life forms within the human host. The extracellular metacyclic promastigote, which is deposited into the skin when the infected sand fly vector takes a blood meal from the uninfected host. The intracellular amastigote stage which resides within a parasitophorous inside macrophages or other phagocytic cells such as neutrophils (Figure 1). Promastigotes are rapidly taken up by phagocytic cells and transform into the intracellular amastigote stage (Clos et al., 2022). Therefore, the prime target antigens for a vaccine candidate are usually associated with the amastigote stage.

## Immunity to *Leishmania*

**Figure 2.**
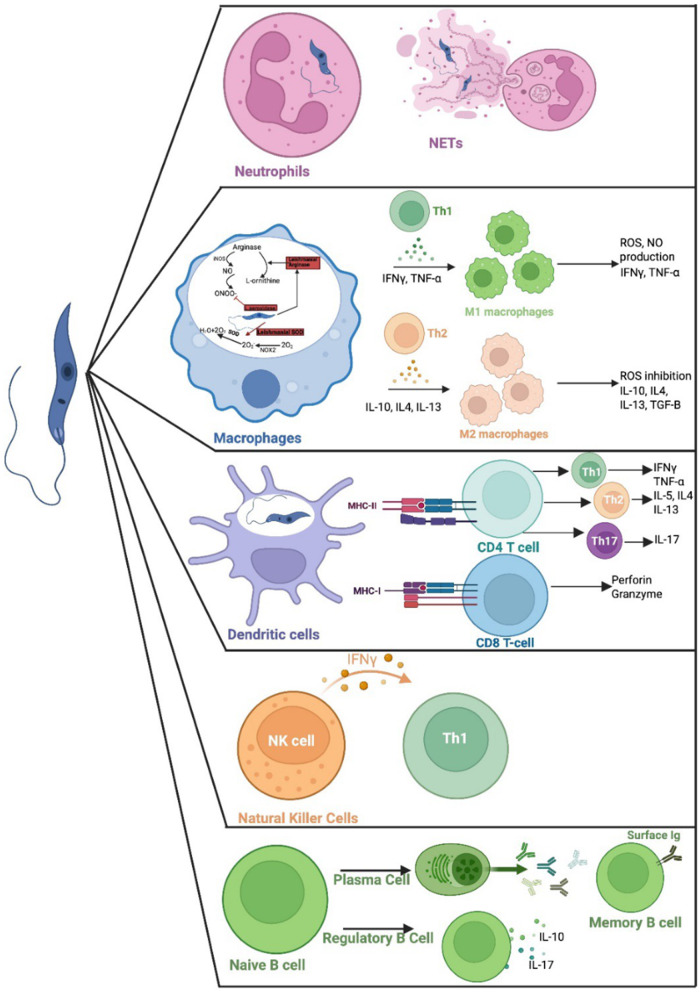
Leishmania parasites impact on immune cells. Neutrophils and parasite interaction results in the release NETs to assist in parasite elimination. Macrophages differentiate to M1 or M2 cells, depending on local immune responses. Stimulated M1 macrophages produce ROS and NO to help eliminate parasites, stimulated M2 macrophages produced cytokines such as IL-3, IL-4 and IL-10 which favour parasite persistence. Dendritic cells are involved in antigen presentation to T cells via MHC class I or MHC class II. Natural killer cells stimulate Th1 production via release of IFN-γ. B cells can produce specific antibody which can promote parasite killing or activated regulatory cells can promote parasite survival via production of anti-inflammatory IL-10 and IL-17 cytokines (created using BioRender.Com software). Figure 2 long description.

*Leishmania* was originally discovered by William Boog Leishman in a patients’ spleen in 1900 (Steverding, 2017). The promastigote form of the parasite that has a flagellum that allows the parasite to move and attach to the gut wall of the female sandfly (Figure 1). Macrophages and other phagocytic cells, e.g. neutrophils or monocytes, take up the parasite, where it resides within a parasitophorous vacuole, and transforms into non-motile intracellular amastigote form (Lodi et al., 2024). Differentiation of parasites from the promastigote to the amastigote form is triggered by the change in pH, temperature, availability of nutrients and gene expression within the mammalian host cell (Pacakova et al., 2022). The amastigote replicates by binary fission, eventually leading to disruption of the host cell and systemic dissemination of the released amastigotes. These parasites can infect new phagocytic cells or be taken up by a sandfly when it takes a blood meal from an infected host. The amastigotes transform into infective promastigotes in the infected sandfly via six developmental stages, eventually developing into infective, metacyclic promastigotes (Yanase et al., 2024). The immune response responsible for protection or susceptibility to *Leishmania* is complex and depends on the infecting species and host factors (Nunes et al., 2025). Different cells of the immune system and their products are involved in determining the outcome of an infection (Figure 2). But ultimately production of specific T cells which can activate killing of intracellular amastigotes within infected macrophages or killing of infected cells, without inducing a damaging inflammatory response, is required. Neutrophils have a key role in immunity to *Leishmania* in the mammalian host, they enhance the inflammation and help parasite elimination via neutrophil extracellular traps (NETs; Paton et al., 2025; Figure 2). Release of NETs is associated with programmed cell death and promotes pro-inflammatory response (Do Nascimento et al., 2025). However, parasites within infected neutrophils can be taken up by macrophages, and escape killing, so that the neutrophil acts as a ‘Trojan horse’ (Paton et al., 2025). Macrophages have different mechanisms to kill pathogens, e.g. release of antimicrobial enzymes, peptides, nitric oxide and super oxide. Macrophages express Phox and NOX2 or type 2 nitric oxide synthase (iNOS or NOS2) which induces production of nitric oxide (NO) and superoxide anion (O2^-,^) which can interact to produce peroxynitrite, which has a higher toxicity (Bogdan et al., 2024). *Leishmania* produces L-ornithine (Goto, 2024) and superoxide dismutase (SOD) homologues to help detoxify these toxic products (Rossi and Fasel, 2018) (Figure 2). Macrophages can be typed as M1 or M2 cells based on the products they produce. M1 macrophages have a pro-inflammatory phenotype and produce interferon gamma (IFN-γ), IL-12 and tumour necrosis factor alpha (TNF-α) (Goto and Mizobuchi, 2023; Saini et al., 2024), which would activate macrophage killing mechanisms and would help to eliminate *Leishmania*. Whereas, M2 macrophages have an anti-inflammatory phenotype and produce IL-4, IL-13, IL-10 and a transforming growth factor beta (TGF-β), cytokines which would inhibit macrophage activation and thus promote parasite survival within macrophages (Ayala et al., 2024) (Figure 2). Induction of M1 or M2 macrophages is regulated by Th1 and Th2 mediated immune response respectively and immunity to Leishmania often depends on the balance of Th1/Th2 responses within the infected host (Saini et al., 2024). Dendritic cells (DCs), located in peripheral tissues, express major histocompatibility complex (MHC) molecules and their activation regulates cytokıne secretion by other immune cells. DCs are one of the first cells to interact with parasites after infection, and they are involved in antigen presentation to CD4^+^ T cells via MHC class II molecules (Tibúrcio et al., 2019). Antigen specific CD4^+^T cells can differentiate into Th1, Th2 or Th17 cells depending on the local conditions. Activation of specific Th1 cells is associated with parasites elimination through the production of the pro-inflammatory cytokines IFN-γ and TNF-a. Activation of specific Th2 cells is linked with the production of IL-4, IL-5, and IL-10, which are related to parasite persistence (Tibúrcio et al., 2019; Paton et al., 2025). Activation of specific Th17 cells can have protective or exacerbate effects depending on when they are activated during infection (Morales-Primo et al., 2024; Figure 2). CD8^+^ T cells recognize peptides presented via MHC I. Once stimulated they secrete cytotoxic granules which can induce programmed cell death of the antigen presenting cell i.e. the infected macrophage, and they produce pro-inflammatory cytokines such as IFN-γ and TNF, which would stimulate macrophage antimicrobial killing mechanisms (Rodrigues et al., 2021). *Leishmania* parasites can activate natural killer (NK) cells and they are the primary source of IFN-γ, causing activation of DCs, neutrophils, macrophages and T cells at the start of a *Leishmania* infection (Alizadeh et al., 2023). Activated B cells produce specific antibodies help to eliminate extracellular parasites and they can act as an APC (Paton et al., 2025). In contrast, activated regulatory B cells secrete IL-10 and IL-17, which can supress Th1 responses and promote Th2 immune response, which would favour survival of *Leishmania* parasites (Cai et al., 2025). The role of a specific immune cell in *Leishmania* infection can depend on when it is activated or where it is produced during infection, for example at the start of an infection or during the chronic stages of infection. For example, IL-4 is often associated with parasite survival in *Leishmania* infections as it can down regulate macrophage killing mechanisms. However, IL-4 has a protective role in *L. donovani* infections, as its presence is essential for the production of granulomas, which are part of the liver curing response. Thus, *L. donovani* infection of IL-4 receptor α^−/−^ deficient mice resulted in increased parasite burdens compared to wild-type mice, and this increase in susceptibility in the absence of IL-4 signalling was associated with increased NOS2 expression and decreased serum gamma interferon levels (Stager et al., 2003). Several recent reviews give an excellent overview of host–parasite interactions and the role of specific immune cells for different *Leishmania* species (Tiwari et al., 2024; Paton et al., 2025). One of the main difficulties in developing a vaccine for leishmaniasis is identifying the appropriate antigen(s) that can induce a protective immune response, and the same antigen(s) may not be suitable for all *Leishmania* species.

## Vaccination strategies against leishmaniasis

Historically vaccines for any disease have been divided into three main types. First-generation vaccines use live-attenuated pathogens or whole-killed pathogens (Khalil, 2018), second-generation vaccines use extracted or purified native proteins from a pathogen and third-generation vaccines use naked plasmid DNA or encapsulated DNA encoding for a pathogen-specific protein (Makarani et al., 2025). These types of vaccines have been used in *Leishmania* studies and details of human studies are summarized in Table 1.

**Table 1.** Human vaccines used in *Leishmania* studies Table 1 long description.

Name	Adjuvant	Status	Sponsor	Major finding	Reference
**First-generation vaccine**					
Autoclaved *Leishmania*	BCG	Phase II-III Completed	Tehran University of Medical Sciences	Vaccination did not produce life-threatening side effects. There was no significant difference between groups in terms of production of IFN-γ and IL-10.	(Satti et al., 2001)
**Second-generation vaccine**					
Leish-111 f	MPL-SE	Phase II Completed	AAHI	IFN-γ production from T cell was induced by vaccination against visceral leishmaniasis.	(Nascimento et al., 2010; Chakravarty et al., 2011)
				IgG antibody response induced after cutaneous leishmaniasis.	
LEISH-F2	MPL-SE	Phase II Completed	AAHI	Showed potential therapeutic effects when combined with the adjuvant against cutaneous leishmaniasis.	(2013)
LEISH-F2	MPL-SE + chemotherapy with sodium stibogluconate	Phase I Completed	AAHI	Vaccination was evaluated in term of safety, efficacy and immunogenicity against cutaneous leishmaniasis.	(2012)
LEISH-F3	GLA-SE	Phase I Completed	AAHI	Vaccination induced antigen-specific Th1 responses. Vaccination was safe and effectively induced IFN-γ production	(Coler et al., 2015)
LEISH-F3	SLA-SE	Phase I Completed	AAHI	Vaccination was safe in humans and induces antigen-speciflc cellular responses.	(2015; Carter et al., 2016)
LEISH-F3	GLA-SE or MPL-SE	Phase I completed	NIAID		
**Third-generation vaccine**					
ChAd63-KH (Leish2a)	None	Phase II completed	University of York	It was observed that the frequency of IFN-γ producing by CD8 + T cells was not significantly different between high and low-dose vaccination treatment.	(Younis et al., 2021)
ChAd63-KH (LEISH2b)	None	Phase II Completed	University of York	Naïve CD8 + T cells and pre-existing memory CD8 + T were elicited by vaccination. There was observed IL-10 mediated regulation of immune response.	(Lacey et al., 2022)

**Figure 3.**
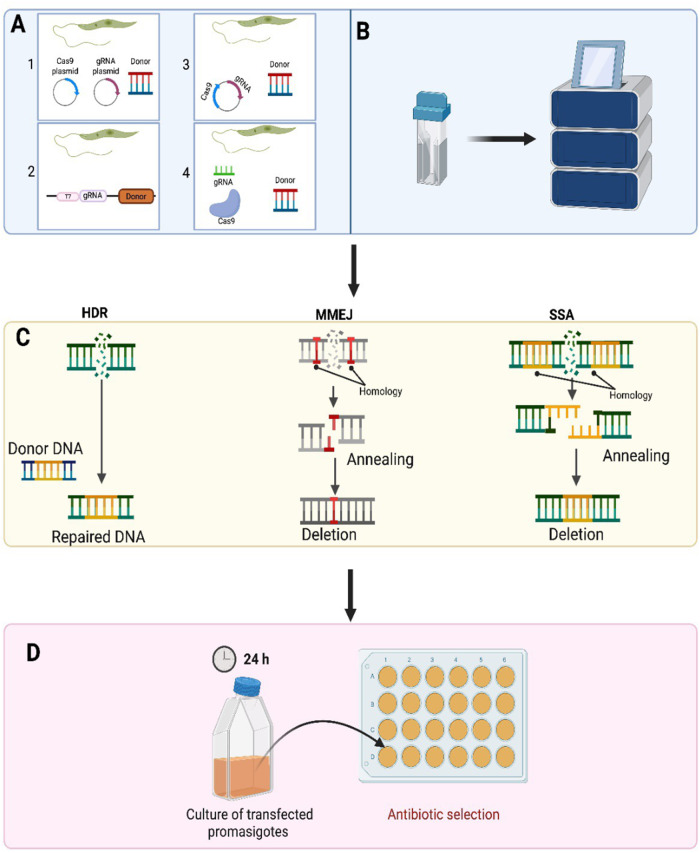
CRISPR/Cas9 is used in Leishmania parasites to either delete or insert different tags to a gene of interest. The various ways were exploited to achieve precise and efficient genome editing via CRISPR-Cas9 in Leishmania parasites. A-1. Strategy using separate plasmids: The sequences encoding Cas9 and gRNA are placed in divergent plasmids. A-2. Single vector use. Both single guide RNA and Cas9 were consolidated in a single vector by Zhang et al. A-3. Introduction of continuous expression system. A CRISPR-Cas9 high-throughput toolkit enabling precise and efficient genome editing was developed by Beneke et al. In 2017. A-4. Vector-free system use. B. Transfection in Leishmania. C. DSB repair mechanisms in Leishmania. Three different DNA repair mechanisms including homology-directed repair (HDR), microhomology-mediated end joining (MMEJ) and single-strand annealing are used for the purpose of DSB repair in Leishmania. Compared to MMEJ, SSA contains longer stretches of homology to repair DSB. D. Antibiotic selection of transfected Leishmania parasites following CRISPR-Cas9 genome editing (created using software from BioRender.Com). Figure 3 long description.

First-generation vaccines are usually more successful as they induce a similar response as wild type parasites. One of the first-generation vaccines used against canine leishmaniasis was the Leishvaccine, prepared using whole-killed *L. amazonesis* promastigotes and Bacillus Calmette–Guérin (BCG) as adjuvant. Immunization with Leishvaccine-induced initial alterations in the innate immune responses mediated by neutrophils and eosinophils. These were followed by modifications in monocyte responses and activation of specific CD4^+^ T cells, CD8^+^ T cells, and B lymphocytes. The vaccine induced a mixed cytokine profile in immunised dogs as both IL-4 (16.7%) and IFN-γ (50%) were produced by the cells of 66.7% of immunized dogs (Araújo et al., 2009; Ayala et al., 2024). It resulted in a 76–80% reduction in parasite burdens in immunized dogs in field studies and stopped immunized dogs infecting sand flies (Borja-Cabrera et al., 2008). Generally first-generation vaccines can be produced at low cost, as they have a simple production process and can easily be distributed worldwide (Ayala et al., 2024). However, these types of vaccines may only induce local inflammation, weak protective immune responses and the vaccines may need cold chain storage, which would increase vaccine costs in endemic countries with high local temperatures and poor refrigeration facilities (Bhattacharya et al., 2024). In some cases, there is a risk that the attenuated parasite could revert to the infective form of the parasite and cause disease symptoms. One way to improve the efficacy of first-generation vaccines is to use Clustered Regularly Interspaced Short Palindromic Repeats/CRISPR-associated protein 9 (CRISPR-Cas9) technology to develop new strains of parasites for the first-generation vaccines (Sharma et al., 2022; Alonso et al., 2023; Seyed et al., 2025). CRISPR-Cas9 genome editing technology has revolutionized genetic engineering (Doudna and Charpentier, 2014), as it provides a simple and efficient way to edit an organism’s chromosomal DNA so that a gene of interest can be inserted or deleted from an organism’s DNA (Zhang and Matlashewski, 2019) (Figure 3).

CRISPR-Cas systems are complex and can be grouped into two classes and six main types. Class I systems use a complex of many as the effector module whereas in Class II systems a single multidomain protein consisting of Cas9, Cas12 or Cas13 is the effector module (Koonin and Makarova, 2019). Among all the identified Cas proteins, the most investigated proteins cuts foreign RNA or DNA directionally, which is why these proteins are known as ‘genetic scissors’ (Gostimskaya, 2022). The CRISPR-Cas9 system was first successfully used by Sollelis et al. (2015) to cause an efficient and precise gene deletion in *Leishmania* parasites (Sollelis et al., 2015). To succeed in CRISPR-Cas9-mediated genome editing in *Leishmania*, the method required the stable expression of a Cas9 nuclease and the presence of a single guide RNA (sgRNA) which has a 20-nucleotide-long protospacer sequence, which is complementary to the targeting sequence. For precise and efficient gene knockout, formation of a complex between Cas9 and the sgRNA is needed. Following the Cas9:sgRNA complex generation, the machinery is guided to the target site, which is found right after the PAM sequence. Subsequently, Cas9 sgRNA complex attaches to the target locus becomes activated, resulting in a double-strand break (DSB) in the organism’s DNA. This DSB takes place 3 nucleotides upstream of the PAM sequence, paving the way for a precise and efficient genome editing (Minet et al., 2018; Zhang and Matlashewski, 2019). In contrast to mammalian cells, *Leishmania* parasites do not possess the most efficacious nonhomologous end-joining pathway to repair the generated DSB (Espada et al., 2021). Instead, *Leishmania* parasites use various homology-mediated repair pathways containing single-strand annealing (SSA) and microhomology-mediated end joining (MMEJ) or homology-directed repair (HDR), helping the donor DNA fragment (gene being inserted) to insert at the deleted region (Zhang and Matlashewski, 2019).

Other approaches have been used to express Cas9 along with sgRNA in *Leishmania* parasites. Sollelis et al. (2015) used the two different plasmids approach detailed in Figure 3A. One plasmid expressed Cas9 nuclease stably under the control of the *Leishmania* DHFR-TS promoter while the other plasmid used to produce sgRNA was under the control of the U6 snRNA promoter and terminator. Electroporation of the two plasmids into *Leishmania* promastigotes produced a knocking out in the tandemly repeated paraflagellar rod 2 locus (PFR2) in *L. major* after a single electroporation round produced no off-target effects. Zhang and Matlashewski (2015) successfully knocked out A2 multigene family (multicopy genes) and the miltefosine transporter *L. donovani* parasites using a single plasmid (Figure 3A) to induce both Cas9 expression and sgRNA transcription under a RNA polymerase I promoter. This strategy was used for point mutation insertion and endogenous gene tagging in *Leishmania* (Bryant et al., 2019).

However, the plasmid-based CRISPR-Cas9 systems conducted by Sollelis et al. and Zhang et al. were time-consuming as they both require plasmid cloning, repeated electroporation steps and extended culture times (especially in Zhang et al.’s approach), Cas9 toxicity and plasmid loss. Beneke et al. (2017) identified a CRISPR-Cas9 high-throughput toolkit to edit kinetoplastids’ genomes precisely and efficiently to overcome these problems (Beneke et al., 2017). In this approach, DNA repair cassettes and all sgRNA DNA templates are produced via PCR using primers designed through an online source named LeishGEdit (http://www.leishgedit.net). It provides one null mutant parasite with 100% efficiency rate from one sgRNA (Moreira et al., 2023). There is no requirement for expensive cloning kits and no need to purchase commercial sgRNAs, making this system much more cost-effective (Beneke et al., 2017; Beneke and Gluenz, 2019). This PCR-based toolkit paves the way for carrying out rapid and precise gene tagging and gene deletion in various kinetoplastid species including *Leishmania.* The PCR-amplified DNA repair constructs and the sgRNA DNA templates are co-transfected directly into parasites, which stably express Cas9 and T7 RNA polymerase, and surviving parasites possessing the desired modifications are produced within 1 week of drug selection. The transfected sgRNA DNA templates are transcribed into the actual sgRNA by T7 RNAP *within the living parasite cells*. Gene deletion or tagging (N-terminal or C-terminal) using various reporters including fluorescent proteins, BirA* and epitope tags can be successfully accomplished using this toolkit (Beneke et al., 2017). Further information on the CRISPR/Cas9 systems used in *Leishmania can* be found in the review by (Abdi Ghavidel et al., 2024). Therefore, the CRISPR/Cas9 system can provide a way of producing a *Leishmania* live vaccine, where parasites can prime the host’s immune system but die before the parasites produce any adverse clinical symptoms. Numerous genetically modified live attenuated *Leishmania* vaccines have been studied but the *centrin* gene-deleted is the most studied. The *L. major* mutant (*LmCen^−/−^*) displayed loss of virulence while inducing robust protective immunity characterized by IFN-γ–producing CD4^+^T cells in BALB/c mice (Zhang et al., 2020). A *L. mexicana* (*LmexCen^−/−^*) live vaccine is only one to ready for human clinical trials. Immunization with *LmexCen^−/−^* promastigotes prepared using CRISPR/Cas9 significantly protected mice and hamsters against *L. donovani* infection, although it did not cause sterile immunity in immunised animals (Karmakar et al., 2022). More recently *Leishmania major* kDNA-associated gene, the universal minicircle sequence binding protein (UMSBP), was edited using CRISPR/Cas9 toolkit developed by Beneke et al. (2017). The single knockout *L. major* parasites (*Lm*UMSBP^+/−^) generated grew significantly less than the wild type in culture and BALB/c infected with this *L. major* strain produced higher levels of IFN-γ and lower levels of IL-4 levels compared to the WT strain. Vaccination with the *Lm*UMSBP^+/−^ strain produce partial protection against *L. major* infection in BALB/c mice (Darzi et al., 2025). Better protection may be achieved using a combination of genetically modified parasites or using a strain that has multiple genetic modification to protect against any chance of the parasite reverting to a virulent form when it circulates in the wild. It has been shown that parasites can form hybrid crosses within the sandfly host so it may not be wise to rely on single genetic changes to reduce parasite virulence (Sádlová et al., 2024). However, perhaps production of sterile immunity is not required for a successful leishmanial vaccine candidate as a recent attempt to identify a product profile for vaccine candidates against *Leishmania* indicated that one that gives significant protection may still be viable (Kaye et al., 2023a).

Second-generation vaccines use extracted and/or purified native *Leishmania* proteins expressed in a suitable vector, such as *Escherichia coli*, plants or a species not infective to humans like *Leishmania tarentolae*. The advantage of these types of vaccines is that there is no probability of the vaccine causing active disease in a vaccinated individual. However, the reduced complexity of the vaccine compared to a whole organism approach means that the vaccine may not induce a protective immune response (Badhwar et al., 2024). These types of vaccines can be produced on a large scale and may be cheap to manufacture (Shital et al., 2024). This may explain why numerous recombinant vaccines have been tested in clinical trials to protect against human leishmaniasis (Abdellahi et al., 2021). But none have resulted in a clinically approved vaccine. Recently, a *Leishmania infantum* kinetoplast-associated protein-like protein (LinKAP) was studied as a vaccine candidate against VL. Immunization with the recombinant LinKAP protein promoted pro-inflammatory responses by inducing enhanced TNF-α and IFN-γ levels and caused a significant reduction in parasite burdens in vaccinated mice and hamsters but did not induce sterile immunity (de Oliveira et al., 2025).

Third-generation vaccines contain naked plasmid DNA or encapsulated DNA in a viral vector (Dinc, 2022). DNA vaccines can provide long-term protection by promoting innate and adaptive immune responses, they can be manufactured easily on a large scale and they can be cheap to produce (Badhwar et al., 2024). The utility of these types of vaccines was demonstrated by the rapid development of COVID vaccines. The main risk associated with DNA vaccines is the possibility that the foreign DNA introduced could integrate into the mammalian genome and causing autoimmune disease or unanticipated side effects (Abdellahi et al., 2021; Bansal et al., 2025). Two of these vaccines has undergone phase II clinical trials for leishmaniasis (Table 1). The most recent *Leishmania* DNA vaccine was the ChAd63K vaccine that encodes the kinetoplastid membrane protein-11 (KMP-11) and hydrophilic acylated surface protein B (HASPB) gene sequences. These antigens are integrated into a chimpanzee adenovirus vector (ChAd63) (Shital et al., 2024). This vaccine has been used in Phase IIb clinical trials, which showed that a single vaccination with ChAd63-KH was safe but did not protect Sudanese patients against PKDL (Younis et al., 2024).

**Figure 4.**
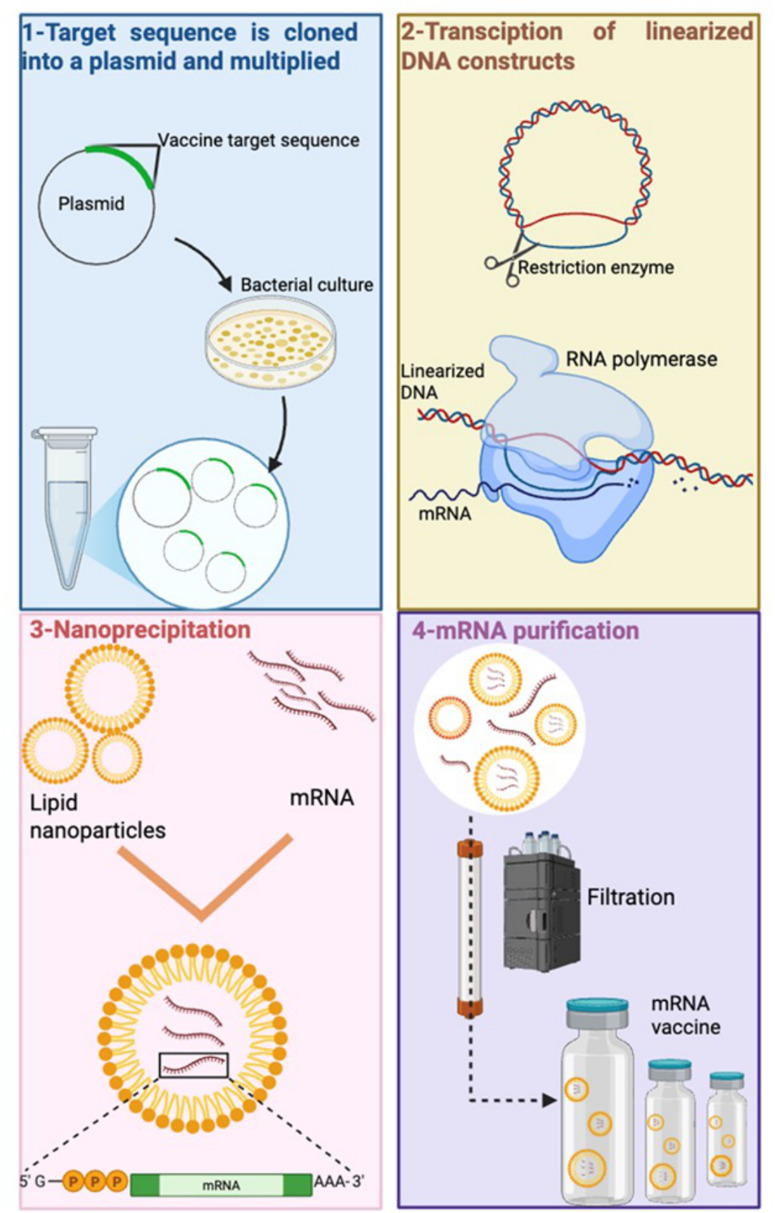
Messenger RNA vaccine production steps used to produce an mRNA vaccine. (1) DNA construct for the vaccine protein is cloned in *E. Coli* bacteria, then amplified; (2) linearized DNA template is transcribed with RNA polymerase and nucleotide triphosphates to obtain mRNA, (3) mRNA carrying poly(A) tail and cap is encapsulated with lipid nanoparticles. (4) The mRNA product is purified to remove any remaining contaminations (created using software provided by BioRender.Com). Figure 4 long description.

RNA vaccine technology offers versatile platform for development of vaccine against infectious diseases, including leishmaniasis (Al Fayez et al., 2023; Figure 4). RNA-based vaccines offered low-cost production, eliciting strong and long-lasting immune response and rapid option to combat complex diseases and were useful in the COVID pandemic (You et al., 2023). Evolution of SARS-CoV-2 variants required the production of a vaccine which can be updated quickly and RNA vaccines provide fast modifications opportunities (Branche et al., 2022). RNA vaccines also remove the need for any culture cell, as the RNA can be produced by *in vitro* transcription, a process which avoids quality and safety issues and requires simple and faster downstream purification to produce mRNA and self-amplifying replicon RNA (repRNA) constructs (Duthie et al., 2022). LEISH-F2 and LEISH-F3 fusion proteins were used to generate an alphavirus-based RNA-expressing the F2 and F3 genes (Duthie et al., 2018). Vaccination with F2-RNA or F2-RNA with the SLA-SE as adjuvant did not protect C57BL/6 mice against *L. donovani* infection compared to controls. However, using a heterologous approach, where mice were immunized with F2-RNA and then boosted with F2 recombinant protein and a synthetic TLR4 ligand formulated in an oil-in-water emulsion (SLA-SE), caused a significant reduction in parasite burdens (Duthie et al., 2018, 2022). This study may indicate that *Leishmania* RNA vaccines need to persist longer *in vivo* to prime antileishmanial immune responses. It is possible to chemically modify RNA to increase it *in vivo* longevity. Studies have shown that natural mRNAs, including 5′-capped and/or 3′-terminated polyadenylated tail structures, have a half-life of 16.4 h in human blood. It is possible to increase the *in vivo* half-life of RNA by up to 30 days by making chemical modifications but there is a danger that the RNA could cause off target effects e.g. cardiotoxicity, a feature shown by Spikevax (mRNA-1273, Moderna) and Comirnaty (BNT162b2, Pfizer/Biontech) vaccines (Boros et al., 2024).

## Administration of a leishmanial vaccine

**Figure 5.**
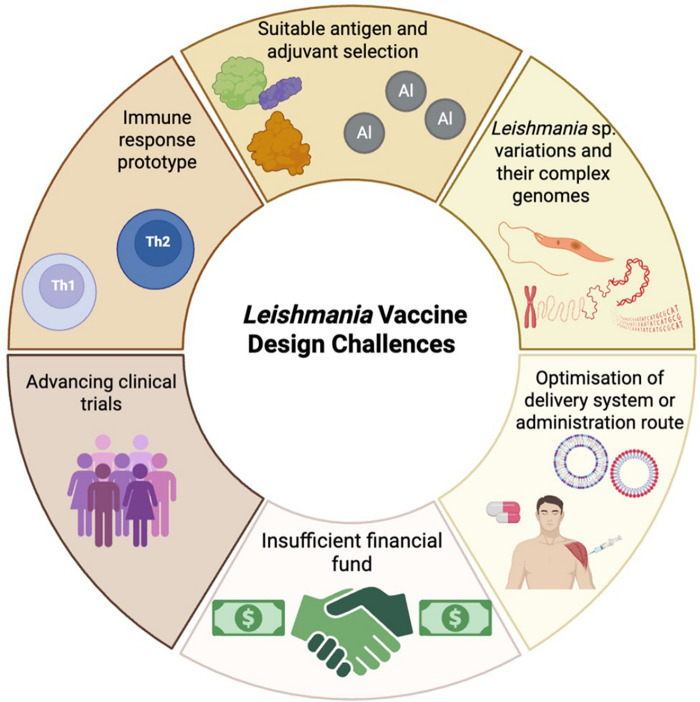
Leishmania vaccine design challenges (created with BioRender.Com). Figure 5 long description.

Once a vaccine candidate for leishmaniasis has been identified there are additional challenges a leishmanicidal vaccine much address (Figure 5). In an ideal world, the vaccine should induce long-lasting and sustain immunity after easy production, be safe and be cost-effective. Currently there is no recommended vaccine for human use (Bhattacharya et al., 2024) and variation in the virulence *Leishmania* spp. and complexity of *Leishmania* genome (Kaye et al., 2021) may make a vaccine suitable for multiple species difficult. Adjuvants can enhance the protective immune induced by a vaccine and numerous adjuvants have been used in leishmanial vaccine formulations for example, cytokines, saponins and TLR agonists (Saini et al., 2024). Selecting the adjuvant that boosts the appropriate CD4^+^ T cell subsets and cytokine profile will be important (de Franca et al., 2024). For example, protection in CL caused by *L. major* is associated with Th1 responses whereas induction of Th2 responses and IL-4 secretion is associated with progression of disease (Elmahallawy et al., 2021). The vaccine dose administered, frequency of dosing and administration route are also very important. Subcutaneous (SC), intramuscular (IM), and transdermal (TD) routes are commonly in vaccination. IM administration is associated with a lower incidence of side effects but it requires trained personnel to immunised individuals and there is a risk of needle-stick injuries (Bouazzaoui and Abdellatif, 2024). Alternative routes can overcome these limitations such as using mucosal, nasal or oral routes (Eom et al., 2024) which would not require use of a needle. Mucosal vaccination can also be improved by using nanocarriers to improve antigen delivery (Dacoba et al., 2017) by improving antigen stability, controlling antigen release and acting as an vaccine adjuvant (d’Amico et al., 2021). Various antigen delivery systems have been used in vaccine research including lipid-based nanoparticles, self-assembled protein nanoparticles, polymeric nanoparticles and virus-like particles (VLPs). Particle size, particle shape, surface charge, rigidity and targeting ligands can affect vaccine efficacy (Chaves et al., 2024). In a recent study, exosomal nanoparticles derived from *Leishmania* containing the immunogenic domains of LACK, TSA, KMP11 and LmSTI1 of *L. major* were evaluated as a vaccine candidate in BALB/c mouse challenged with *L. major*. Immunization enhanced IFN-γ production but had no effect on lesion (Sangani et al., 2025).

Oral vaccines have been used to protect against other types or infection for example, oral rotavirus vaccines, oral cholera vaccine and oral typhoid vaccine (Kwong et al., 2023). Pre-clinical hamster studies showed that oral administration of HAdV-5 expressing the spike protein of SARS-CoV-2 protected against SARS-CoV-2 infection (Langel et al., 2022). Comparison of subcutaneous, oral, and intranasal vaccination with *L. infantum* K39 kinesin antigenic protein and hydrophobin partner fusion vaccine in female BALB/c mice showed that intranasal and oral routes were better at inducing specific IgG production, indicating that mucosal administration can generate a systemic immune response (Silva et al., 2023).

Clinical trials of any leishmaniasis vaccine candidate are challenging due to lack of funding, public health value and researchers’ reluctance (Kaye et al., 2021). One way to minimise risk of product investment and give an efficient protocol for clinical trials is to use a ‘controlled human infection model’ to improve the understanding of disease pathogenesis and host immune responses (Laurens, 2025). This approach was used with 14 participants infected with *L. major* on 24 January-12 August 2022 (2023). The clinical, parasitological and immunological data obtained from participants may help to select vaccine candidates for further clinical research (Parkash et al., 2024).

## Conclusion

Leishmaniasis has been a burden on humanity for many years and identification of a clinical vaccine would have a major impact on human health. Poverty, malnutrition, low income, drug resistance and poor access to effective drugs all contribute to an inability to eliminate this disease (Paliwal et al., 2021; Bamorovat et al., 2023, 2024). Leishmanization has been used in the Middle East as a traditional medicine, where a person is given an intradermal inoculation of live *L. major* parasites in a hidden site, gives protective immunity to re-infection and prevents a person does developing a disfiguring cutaneous lesion (Mohebali et al., 2019). Therefore, there is precedence to suggest that a live vaccine candidate may have the best chance of success. Using recently developed molecular techniques it should be possible to produce a live vaccine strain, with multiple genetic alterations so it cannot revert to a virulent form. In addition, the variant must not cause clinical symptoms after infection, be easy to grow *in vitro* and needs to be given as a single dose. Current research characterizing genetic mutants not only give valuable for understanding the basic biology of *Leishmania* but may also identify useful vaccine candidates.

## Figure 1 Long description

SAND FLY STAGE section with four boxed drawings connected by arrows around a sandfly. Top left box shows labels: Procyclic promastigote and a right pointing arrow labeled Metacyclogenesis leading to Metacyclic promastigote. Top right box shows multiple elongated parasite shapes and the text: Sand fly injects promastigotes when it takes blood meal. A diagonal arrow points down toward a central sandfly drawing. Bottom right box shows the text: Amastigotes infected macrophages. A vertical arrow points upward from this box toward the sandfly. Bottom left box shows the text: Amastigotes release in the midgut. A vertical arrow points upward from this box toward the top left box. Between the sandfly stage and the human stage, a human torso and arm are shown with a sandfly at the skin. A vertical arrow points downward from the sandfly stage toward the human stage. HUMAN STAGE section with three stacked boxes connected by arrows. Top box shows the text: Macrophages phagocyte promastigotes, with drawings of macrophages and elongated parasite shapes. A downward arrow leads to the middle box. Middle box shows the text: Promastigotes transform into amastigotes inside macrophages and a label inside the box reading: Temperature and Gene expression changes. A downward arrow leads to the bottom box. Bottom box shows the text: Amastigotes multiply and infect other immune system cells, with drawings of multiple small round forms and several immune cell shapes.

Navigate back to Figure 1.

## Figure 2 Long description

The diagram illustrates the interactions of various immune cells with Leishmania parasites. Neutrophils release NETs. Macrophages differentiate into M1 and M2 types, with M1 producing ROS and NO and M2 producing IL-10, IL-4, IL-13 and TGF-β. Dendritic cells present antigens to CD4 T cells via MHC II and to CD8 T cells via MHC I. CD4 T cells differentiate into Th1, Th2 and Th17 cells, producing IFN-γ, TNF-α, IL-4, IL-5, IL-13 and IL-17. CD8 T cells release perforin and granzyme. Natural Killer cells release IFN-γ, stimulating Th1 cells. Naïve B cells differentiate into plasma, regulatory and memory B cells, with regulatory B cells producing IL-10 and IL-17.

Navigate back to Figure 2.

## Figure 3 Long description

The diagram illustrates the CRISPR-Cas9 genome editing process in Leishmania parasites. In section A, four strategies are depicted: 1 shows separate plasmids for Cas9 and gRNA; 2 consolidates both in a single vector; 3 introduces a continuous expression system; 4 uses a vector-free system. Section B depicts the transfection process, showing a pipette transferring material to a stack of containers. Section C details DNA repair mechanisms: HDR uses donor DNA to repair, MMEJ involves homology and annealing leading to deletion and SSA uses longer homology stretches for deletion. Section D shows the culture of transfected promastigotes over 24 hours, followed by antibiotic selection in a multi-well plate.

Navigate back to Figure 3.

## Figure 4 Long description

The diagram illustrates the mRNA vaccine production process in four steps. Step 1: 'Target sequence is cloned into a plasmid and multiplied.' It shows a vaccine target sequence inserted into a plasmid, followed by bacterial culture multiplication. Step 2: 'Transcription of linearized DNA constructs.' It depicts the use of a restriction enzyme and RNA polymerase to transcribe linearized DNA into mRNA. Step 3: 'Nanoprecipitation.' This involves mRNA being encapsulated with lipid nanoparticles. Step 4: 'mRNA purification.' The mRNA is filtered to produce the final mRNA vaccine, shown in vials.

Navigate back to Figure 4.

## Figure 5 Long description

The diagram presents challenges in designing a Leishmania vaccine. At the center is 'Leishmania Vaccine Design Challenges.' Surrounding it are sections: 'Suitable antigen and adjuvant selection' with images of antigens and adjuvants, 'Leishmania sp. variations and their complex genomes' with DNA illustrations, 'Optimisation of delivery system or administration route' with a person receiving a vaccine, 'Insufficient financial fund' with handshake and money symbols, 'Advancing clinical trials' with a group of people and 'Immune response prototype' with Th1 and Th2 cells depicted.

Navigate back to Figure 5.

## Table 1 Long description

The table lists human vaccine candidates studied for leishmaniasis, organized by vaccine generation, and summarizes adjuvant used, clinical phase/status, sponsor, major findings, and citations. A first-generation whole-parasite vaccine (autoclaved Leishmania with BCG; Phase II–III, Tehran University) reported no life-threatening side effects and no significant between-group differences in IFN-γ or IL-10. Second-generation recombinant vaccines sponsored mainly by AAHI (Leish-111f, LEISH-F2, and multiple LEISH-F3 formulations) were completed through Phase I–II and generally induced antigen-specific cellular/Th1 responses, including IFN-γ production; LEISH-F2 is also noted for IgG responses and potential therapeutic benefit when paired with adjuvant, and one LEISH-F2 study combined vaccination with sodium stibogluconate chemotherapy. LEISH-F3 trials using GLA-SE or SLA-SE reported safety and antigen-specific cellular responses, while one NIAID-sponsored LEISH-F3 entry (GLA-SE or MPL-SE) has no major finding recorded in the table. Third-generation viral-vectored ChAd63-KH candidates (University of York; Phase II) reported CD8+ T-cell responses: one study found no significant difference in IFN-γ–producing CD8+ T cells between high and low doses, and another reported elicitation of naïve and memory CD8+ T cells with IL-10–mediated regulation. Overall, the entries emphasize immunogenicity and safety outcomes more than definitive clinical protection, and several findings are qualitative or incomplete as presented.

Navigate back to Table 1.
